# Antioxidant Properties and Secondary Metabolites Profile of *Hyptis colombiana* at Various Phenological Stages

**DOI:** 10.3390/molecules28196767

**Published:** 2023-09-22

**Authors:** Sheila B. Beltrán, Lady J. Sierra, José L. Fernández-Alonso, Angie K. Romero, Jairo R. Martínez, Elena E. Stashenko

**Affiliations:** 1 Centro de Investigación en Biomoléculas-CIBIMOL, Laboratorio de Cromatografía y Espectrometría de Masas-CROM-MASS, Universidad Industrial de Santander, Bucaramanga 680002, Colombia; sheila2218097@correo.uis.edu.co (S.B.B.); lady.sierra2@correo.uis.edu.co (L.J.S.); cenivam.sgr3@uis.edu.co (A.K.R.); jmartine@uis.edu.co (J.R.M.); 2 Real Jardín Botánico—CSIC, Claudio Moyano 1, 28014 Madrid, Spain; jlfernandeza@rjb.csic.es

**Keywords:** *Hyptis colombiana*, *Cantinoa*, essential oils, extracts, antioxidant activity, phenological stage

## Abstract

*Hyptis colombiana* (Lamiaceae family), a species also treated as *Cantinoa colombiana* in a recently segregated genus from *Hyptis*, is a perennial herb or subshrub native to the Andes of northern South America. *H. colombiana* leaves are commonly used in traditional medicine to treat respiratory and digestive illnesses. In this study, *H. colombiana* plants at different phenological stages (vegetative, flowering, and post-flowering) were harvested to obtain essential oils (EOs) and extracts (from fresh plant materials or post-distillation waste) whose chemical compositions and antioxidant activities were determined. *H. colombiana* EOs distilled by microwave-assisted hydrodistillation were analyzed by GC/MS/FID, and hydroalcoholic extracts obtained from fresh plant materials or post-distillation waste were analyzed by UHPLC-ESI^+/−^-Orbitrap-MS. The antioxidant activity was evaluated by the ABTS^+•^ and ORAC assays. The principal compounds found in EOs were sesquiterpene hydrocarbons (65%); specifically, (*E*)-β-caryophyllene and germacrene D. Pyranone, rosmarinic acid, rutin, and *p*-hydroxybenzoic acid were the main constituents in *H. colombiana* extracts. After analyzing the chemical composition and antioxidant activity (ORAC) of EOs and hydroethanolic extracts from flowering *H. colombiana* plants, minimal variations were found. It is advisable to harvest *H. colombiana* plants during their flowering stage to acquire EOs and extracts that can be utilized in the agro-industry of EOs and their natural derivatives.

## 1. Introduction

In recent years, the trend of consuming natural ingredients in final products has increased. This has generated a rise in the demand for essential oils (EOs) and plant extracts as natural components in food [1,2,3]. Every year, in India, around six million tons of plant materials residue result from the distillation process of geranium (*Pelargonium graveolens*), lemongrass (*Cymbopogon flexuosus*), citronella (*C. winterianus*), palmarosa (*C. martinii*), and mint (*Mentha arvensis*) [4].

The distillation of aromatic and medicinal plants produces EO, hydrolate, and residual biomass. After their distillation, the plant materials are used for animal feed [5], composting [6], or biofuel [7]; however, these post-distillation residues may contain potentially useful bioactive molecules, such as phenolic compounds, with antioxidant [8] and antimicrobial [9] properties.

In 2022, the EO market was valued at USD 8.8 billion, having the highest contributions in the food, medicine, cleaning products, and aromatherapy sectors [10]. In Colombia, the import of EOs (USD 15.11 million, 2022) as input for industries was higher than the national production of EOs (USD 0.61 million, 2022) [11].

Colombia is home to a wide variety of plant species that researchers study to identify those with potential uses in various industries. One group of plants, the *Hyptis* Jacq. s.l. (Lamiaceae) genus, along with related satellite genera, like *Hyptidendron* Harley and *Condea* Adanson, as well as recently separated ones, like *Cantinoa* Harley and J. Pastore (part of the subtribe Hyptidinae Endl.), are naturally found in tropical regions of the Americas and are quite prevalent [12,13].

Among these plants, the *Hyptis* genus, which includes various species, is the second most diverse group in Colombia, right after the *Salvia* L. genus. In Colombia alone, there are 43 recognized variations within the *Hyptis* genus [14,15]. One of these species is *H. colombiana*, a perennial herb or aromatic subshrub that originates from the Andes in northern South America. It can be found growing across a significant portion of Colombia, thriving at altitudes between 600 and 2700 m [15,16]. This plant is characterized by its upright, branching flower stems that can reach lengths of 60 to 70 cm. Its flowers are densely packed in compact spiky arrangements [12].

Although some authors have referred to this species as *Cantinoa* colombiana (Epling Harley and J. Pastore), placing it in a separate group from the *Hyptis* genus, we have chosen to use its original and more established name within the *Hyptis* genus in this work. This decision was made because a comprehensive review of the entire group is still pending [13,17]. Plants belonging to the *Hyptis* genus (referred to as *Hyptis* s.l.) have been traditionally used for treating respiratory ailments and stomach and intestinal issues and as agents against bacteria. They also possess properties that combat microbes and act as antioxidants [17,18]. The most commonly found compounds in EOs and extracts from *Hyptis* spp. plants include germacrene D, (*E*)-β-caryophyllene, rosmarinic acid, caffeic acid, chlorogenic acid, rutin, quercetin, and luteolin [19,20,21,22,23,24,25,26,27,28].

The antioxidant properties of EOs and extracts obtained from plants depend on their phytochemical constituents [29,30], which vary according to the agronomic conditions of growth and development of the crop, its geographical location, and the phenological stage and maturity of the plant, among others [31]. Until now, no publication has been found on the chemical composition and antioxidant activity of EOs and extracts of *H. colombiana* collected at its different phenological stages. Knowledge of the changes in the secondary metabolite profiles during plant development is very important for the standardization of its cultivation for commercial uses.

The plant materials in the vegetative, flowering, and post-flowering stages were collected between October 2021 and January 2023 from an experimental plot with 12 *H. colombiana* plants, previously established from the accessions obtained in field outings in the Department of Santander, Colombia. The objective of this work was to identify the favorable phenological stage for the harvest of *H. colombiana* plants. This would help in reducing alterations in the chemical composition and antioxidant activity of their EOs and extracts.

## 2. Results

### 2.1. Distillation and Extraction Yields

Table 1 shows the yields of EOs distilled by microwave-assisted hydrodistillation (MWHD) and the hydroalcoholic extracts obtained from fresh or post-distillation plant materials. The plant aerial parts were collected at the three phenological stages, i.e., vegetative, flowering, and post-flowering.

The yields of EOs and hydroalcoholic extracts obtained from dried plant materials of *H. colombiana* before and after its distillation were similar for plants collected at three phenological stages (Table S1). The yields of the EOs obtained were higher than those reported by Flores et al. [19] (0.4% (*w*/*w*)) for plants grown in Venezuela. This may be due to the different climatic and geographical conditions in which the plants grew.

The ethanolic extraction yields from aerial parts of fresh (undistilled) *H. suaveolens* reported by Mandal et al. [32] (2.64% *w*/*w*) and Medoatinsa [33] (8% *w*/*w*) were lower than those obtained from *H. colombiana*. No reports were found on hydroalcoholic extracts of *H. colombiana* plants before or after their distillation or on their variation within the phenological stages.

### 2.2. Analysis of EOs from H. colombiana Plants Collected at Different Phenological Stages

**Figure 1:**
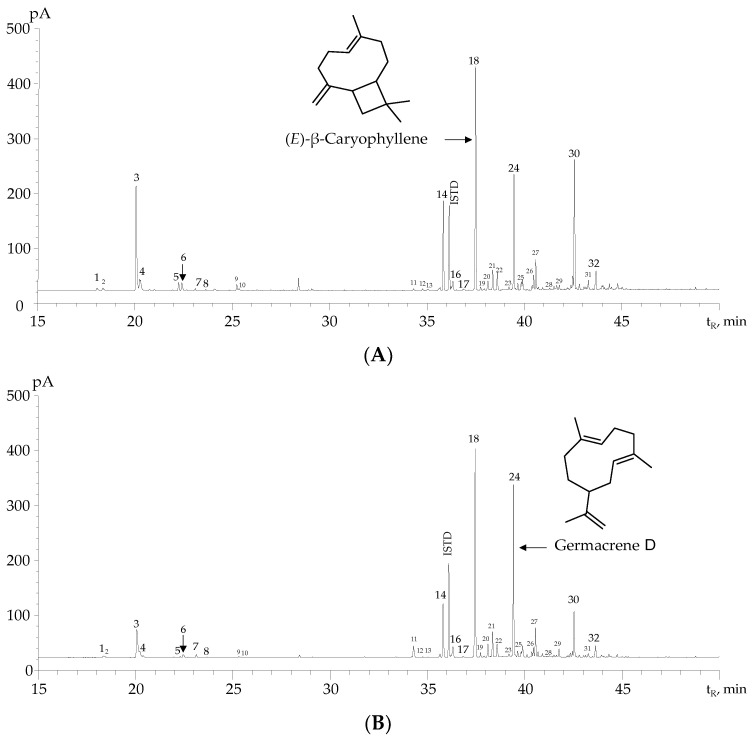

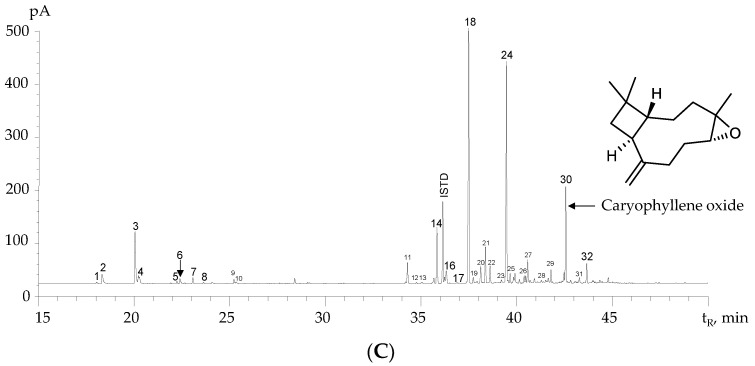
Chromatographic profiles obtained by GC/FID of the *H. colombiana* EOs distilled from plants collected at three phenological stages. (**A**) Vegetative. (**B**) Flowering. (**C**) Post-flowering. DB-5MS column (60 m), split 1:30. See the identification of chromatographic peaks in Table 2.

The chromatographic profiles of the EOs obtained by MWHD from *H. colombiana* plants harvested at three different phenological stages are displayed in Figure 1. Thirty-two compounds (relative amount >0.1%) found in these EOs are presented in Table 2 with their experimental linear retention indices (LRIs) and those found in the literature [34,35,36] for two chromatographic columns with polar and nonpolar stationary phases.

Twenty-eight terpenes, representing 90% of the EO compounds, were positively identified. The study of the fragmentation patterns, reflected in their mass spectra, allowed the tentative identification of two sesquiterpene hydrocarbons (Figures S1 and S2 in Supplementary Materials) and two oxygenated sesquiterpenes (Figures S3 and S4).

Table S2 shows the analytical figures of merit measured for the standard substances used for the quantification of compounds present in the *H. colombiana* EOs. The external standard calibration method was used to quantify the identified compounds with relative GC peak areas ≥ 2% in the chromatograms of *H. colombiana* EOs (Table S3).

### 2.3. Analysis of H. colombiana Hydroethanolic Extracts Using UHPLC-ESI^+/−^-Orbitrap-MS

**Figure 2:**
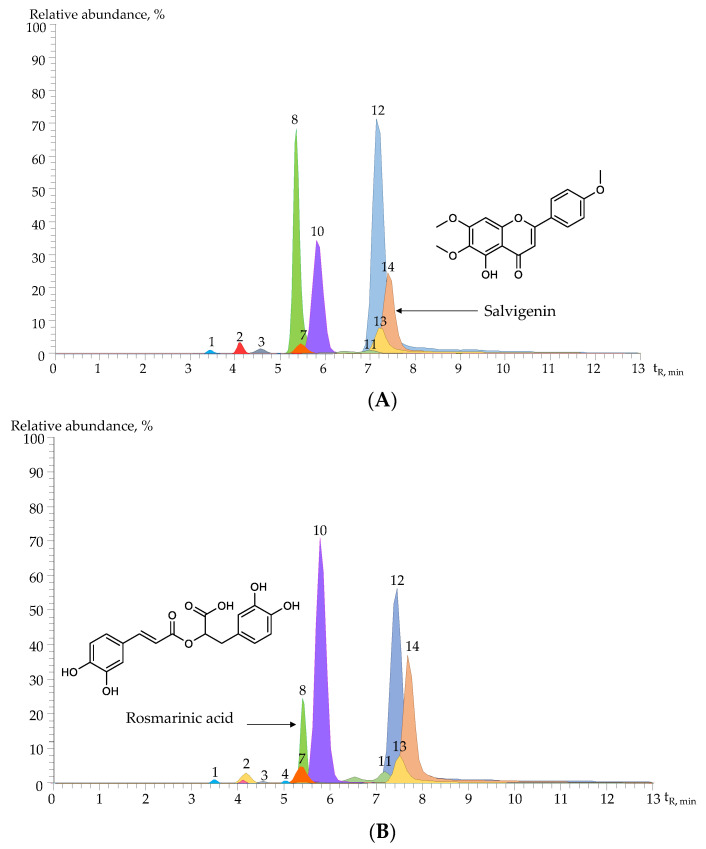

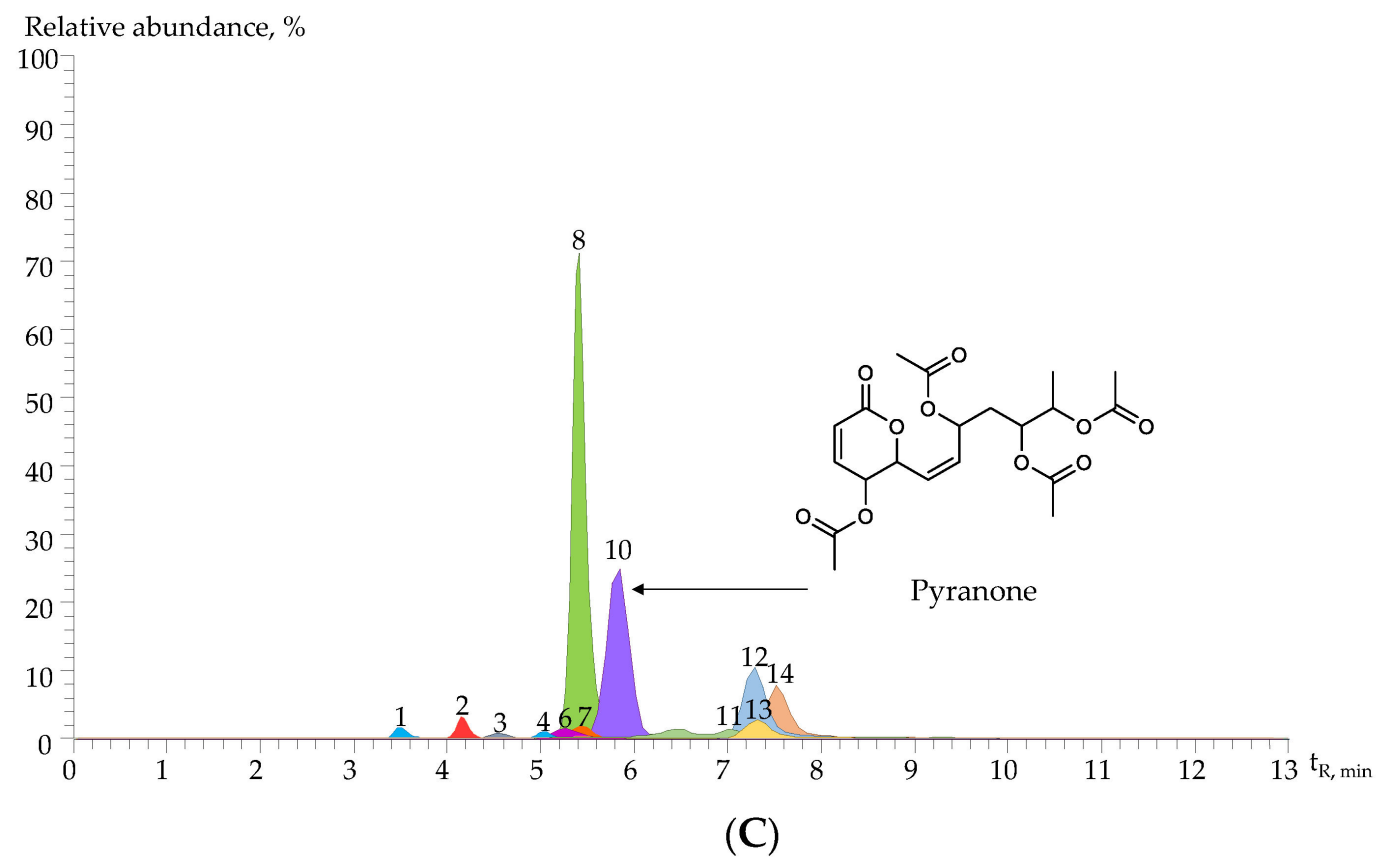
Extracted ion currents (EICs) of protonated [M+H]^+^ or deprotonated [M–H]^−^ molecules, obtained by UHPLC-ESI^+/−^-Orbitrap-MS (scale 6.2 × 10^7^), of compounds in the hydroalcoholic extracts of *H. colombiana*, isolated from fresh plant materials at different phenological stages (**A**) Vegetative stage. (**B**) Flowering stage. (**C**) Post-flowering stage. See compound identification in Table S5.

Table S5 shows the exact experimental masses, the measurement error (∆ppm < 3), and the product ions observed in the mass spectra of the identified compounds. Confirmatory identification was performed by comparing mass spectra and experimental retention times with those of standard compounds. The tentative identification was based on the study of the fragmentation patterns and data reported in the scientific literature. Figure 2 and Figure S5 show the extracted ion currents (EICs) of protonated [M+H]^+^ or deprotonated [M-H]^−^ molecules, depending on the analyte, obtained by UHPLC-ESI^+/−^-Orbitrap-MS operated in dual acquisition mode for both positive and negative ions filtered previously in selected ion monitoring (SIM) mode.

Fourteen compounds were identified in the hydroalcoholic extracts obtained before and after the distillation of *H. colombiana* aerial parts. These compounds include six flavones (apigenin-*C*,*C*-diglucoside, luteolin-*C*-hexoside-*O*-desoxyhexosyl, luteolin-7-*O*-glucoside, hydroxylated salvigenin, trihydroxy-trimethoxyflavone, and salvigenin), four hydroxycinnamic acids (*p*-hydroxybenzoic, caffeic, *o*-hydroxybenzoic, and rosmarinic acids), two flavonols (rutin and kaempferol-3-*O*-rutinoside), a sesquiterpene lactone, and a pyranone (Table S5).

A pyranone (compound N° 10, Table S5) was detected; product ions generated by [M+H]^+^ fragmentation appeared at *m*/*z* 367.13846, [(M+H)–C_2_H_4_O_2_]^+^ and at *m*/*z* 325.12781, [(M+H)–C_2_H_4_O_2_–C_2_H_2_O]^+^. Some authors [46,47] have described different furanones and pyranones (pectinolides) present in *H. pectinata* (L.) Poit. and *H. monticola* Mart. ex Benth. Boalino et al. [46] characterized pectinolide E [C_20_H_26_O_10_, M^+●^ *m*/*z* 426] by NMR and GC/MS. The authors reported that in the mass spectrum of pectinolide E, product ions appeared at *m*/*z* 366, 306, 237, and 204. Based on these MS data, a pyranone, C_20_H_26_O_10_, was tentatively identified in the extracts of *H. colombiana* under study with [M+H]^+^ at *m*/*z* 427.16000 and the abovementioned characteristic fragments (Figure S6).

Table S6 contains information on the quantification of the 14 compounds identified in the hydroalcoholic extracts obtained from *H. colombiana* plants before and after their distillation. The quantification parameters obtained from the external calibration method using standard compounds are reported in Table S2.

### 2.4. Antioxidant Activity of H. colombiana EOs and Extracts

Table 3 presents the antioxidant activity evaluation of the EOs and the hydroalcoholic extracts of *H. colombiana* and individual terpene and phenolic compounds identified in the *H. colombiana* EOs and extracts. These values were evaluated using the 2,2′-azino-bis(3-ethylbenzothiazoline-6-sulfonic acid) radical cation (ABTS^+•^) and oxygen radical absorption capacity (ORAC) assays.

## 3. Discussion

### 3.1. Chemical Composition of H. colombiana EOs

The EOs of *H. colombiana* contain mainly sesquiterpene and monoterpene hydrocarbons. The major compounds of the EOs of the plants collected at the vegetative, flowering, and post-flowering stages were sabinene, (*E*)-β-caryophyllene, germacrene D, and caryophyllene oxide. The concentrations of (*E*)-β-caryophyllene (230 mg substance/g of EO) and germacrene D (200 mg substance/g of EO) in the EOs distilled from plants gathered at vegetative, flowering, and post-flowering stages were not significantly different (Table S4); however, the highest variations were observed for sabinene in all EOs distilled from plants harvested at the three phenological stages (Table S4).

Flores et al. [19] studied the chemical composition of EOs from *H. colombiana* in Venezuela and reported (*E*)-β-caryophyllene (29.5%), germacrene D (22.2%), and caryophyllene oxide (3.5%) as major compounds. The authors [19] found that EOs of *H. colombiana* cultivated in Venezuela have antibacterial properties against *Staphylococcus aureus* and *Enterococcus faecalis*. 

The main ingredient in the essential oils of *H. colombiana* is (*E*)-β-caryophyllene, a compound produced by many plants to protect themselves from plant-eating animals. This is achieved by attracting the creatures that naturally prey on those herbivores [48]. Inside the plant cells, (*E*)-β-caryophyllene is created in the cytosol by changing farnesyl diphosphate into a farnesyl carbocation, which then forms an 11-membered bicyclic hydrocarbon [49]. The United States Food and Drug Administration (FDA) has approved the use of (*E*)-β-caryophyllene as a food additive [1]. This ingredient is also utilized in the perfume industry to add a woody scent [50]. Researchers Dahham et al. [51] extracted (*E*)-β-caryophyllene from the essential oil of *Aquilaria crassna* Pierre ex Lecompte (Thymeleaceae) and found that it had strong antibacterial properties against *Staphylococcus aureus* (with a minimum inhibitory concentration of 3 μM), performing better than the reference antibiotic kanamycin (with a minimum inhibitory concentration of 8 μM). The compound also displayed activity against various fungi, including *Trichoderma reesei*, *Penicillium citrinum*, and *Aspergillus niger* [51]. Given that (*E*)-β-caryophyllene is the major component of the essential oil of *H. colombiana*, this plant could serve as an important source of this compound.

### 3.2. Chemical Composition of H. colombiana Extracts

A pyranone, rosmarinic acid, rutin, and *p*-hydroxybenzoic acid were the major compounds detected in the hydroalcoholic extracts obtained from *H. colombiana* plants before and after distillation (Table S6). Until now, no studies have been found on the chemical composition of hydroalcoholic extracts from *H. colombiana* plants. In the extracts of *H. pectinata* [24] and *H. suaveolens* [25,26], rutin and caffeic and rosmarinic acids were also found. Luteolin-*O*-glucoside, salvigenin, and kaempferol-3-*O*-rutinoside were also present in the *H. colombiana* extracts.

The main component of the hydroalcoholic extracts of *H. colombiana* plants was a pyranone (70 mg/g extract), found in similar amounts in all extracts isolated from plants collected at the three phenological stages. Santana et al. [52] evaluated the cytotoxicity against MDA-MB-231 breast tumor cell lines of ethanolic extracts of *H. pectinata* and of three isolated compounds (pectinolide E, pectinolide J, and hyptolide). Extracts from *H. pectinata* plants inhibited cancer cell proliferation with an IC_50_ of 45.91 ± 0.02 μg/mL. Individual compounds (namely, pectinolide E (85.2 μg/mL), pectinolide J (76.8 μg/mL), and hyptolide (73.6 μg/mL)) had lower inhibition in these cells. However, their mixture increased the inhibition of the cell lines at an IC_50_ of 36.8 μg/mL, indicating a possible synergistic effect.

The amounts of rosmarinic acid and rutin in hydroethanolic extracts from *H. colombiana* plants remained consistently steady, at 17 mg/g and 8 mg/g of dry extract, respectively (see Tables S6 and S7). This remained true regardless of the plant’s growth stage or the method of distillation applied, even though distillation processes are usually associated with causing the breakdown of compounds in the remaining plant material.

Rosmarinic acid is present in plants of the Lamiaceae family, mainly in those of the Nepetoideae subfamily [53]. Rosmarinic acid is biosynthesized in plants from two amino acids, L-tyrosine and L-phenylalanine, through a reaction catalyzed by eight enzymes [54]. The function of rosmarinic acid in plants is to provide resistance to environmental stress and defense against pathogens and herbivores [55]. Furthermore, this compound has antimicrobial [56], antioxidant [57], anti-inflammatory [58], antimutagenic, and antiviral [54] properties.

Rosemary (*Salvia rosmarinus* Schleid) contains high levels of rosmarinic acid (24 mg/g) [59], which is approved by the European Union as a food preservative [60,61]. From every 100 g of *H. colombiana* dry plant material obtained before or after its distillation, up to 200 mg of rosmarinic acid could be isolated. This adds value to the residual plant material after its distillation because it can be used as a source of rosmarinic acid and as a possible food preservative.

### 3.3. Antioxidant Activity of H. colombiana EOs and Extracts 

Many assays have been developed to evaluate the antioxidant capacity of vegetal extracts and other ingredients. Their reproducibility has been a frequent topic of discussion; after 25 years of use by many researchers for different applications, many recommendations have been formulated to obtain reliable results [62]. Kevers et al. performed the validation of the ORAC assay [63] and Xiao et al. published a detailed guideline for the execution of the most common assays [64].

The antioxidant activity values (μmol Trolox^®^/g sample) of the EOs of *H. colombiana* evaluated by the ABTS^+•^ assay presented significant differences (*p* > 0.05) for plants collected at the three phenological stages (Table S8). The highest values were obtained for EOs distilled from plants in the post-flowering stage (904 ± 2 μmol Trolox^®^/g sample). It is possible that the antioxidant activity of the EOs is a result of a combination of various compounds, including caryophyllene oxide, sabinene, and (*E*)-β-caryophyllene. The individual antioxidant activities of these compounds were low, but together they may have a synergistic effect; however, the antioxidant activity values of the extracts obtained from plants collected at the three phenological stages did not show significant differences for the ORAC assay results (Table S8). The ORAC assay examines the antioxidant capacity of radical quenching mediated by the hydrogen transfer mechanism, while the ABTS assay also includes the electron transfer mechanism [65]. Therefore, the differences in antioxidant capacity of the EOs and extracts examined can be attributed to the differences in their content of substances that use electron transfer for interaction with radicals. The main compounds in the extracts of the *H. colombiana* plants collected at different vegetative stages (namely, a pyranone, rosmarinic acid, and rutin), did not have significant variations in their concentrations. *H. colombiana* hydroethanolic extracts exhibited lower antioxidant activity compared to the standard substances of rutin and rosmarinic acid. It is possible that these compounds are the primary contributors to the antioxidant activity found in the extracts. Other extract constituents that may contribute to the total antioxidant capacity are hydroxycinnamic acids and flavones.

The antioxidant activity of *H. colombiana* extracts has not been previously reported, but studies have been found on other species of the genus *Hyptis* spp. [65,66]. Tafurt-García et al. [66] evaluated the antioxidant activity measured by the ABTS^+•^ assay of ethanolic extracts of *H. dilatata* Benth. (903 ± 64 mmol Trolox^®^/kg extract) and *H. conferta* Benth. (721 ± 27 mmol Trolox^®^/kg extract). Dos Santos et al. [67] determined the antioxidant activity of ethanolic extracts from various plants of the genus *Hyptis* spp., including *H. campestris* Harley and J. Pastore, by ABTS^+•^ (290 mg Trolox^®^/g sample equivalent to 1158 μmol Trolox^®^/g sample) and ORAC (4516 μmol Trolox^®^/g sample) assays. The antioxidant activity values measured in this work were similar to those reported by the aforementioned authors [66,67] for other *Hyptis* spp.

## 4. Materials and Methods

### 4.1. Chemical Substances and Reagents

The standard substances α-pinene (98%), sabinene (95%), *p*-cymene (99%), limonene (97%), linalool (97%), γ-terpinene (96%), (*E*)-β-caryophyllene (98.5%), germacrene D (90%), α-humulene (96%), caryophyllene oxide (95%) (98%), *p*-hydroxybenzoic acid (99%), caffeic acid (98%), *o*-hydroxybenzoic acid (99%), rosmarinic acid (97%), rutin (94%), AAPH (97%), and fluorescein (99%) were purchased from Sigma Aldrich (St. Louise, MO, USA), and luteolin-7-*O*-glucoside (98%), kaempferol-3-*O*-rutinoside (98%), and salvigenin (98%) were purchased from ChemFaces (Wuhan, China); vitexin (≥95%) was purchased from PhytoLab GmbH (Vestenbergsgreuth, Bavaria, Germany). Solvents and other reagents used in the extraction process, GC/FID grade dichloromethane, ammonium formate (99%), 2,2′-azino-bis(3-ethylbenzothiazoline-6-sulfonic acid) (ABTS), formic acid HPLC grade, potassium persulfate (99%), and absolute ethanol (96%) were purchased from Merck (Darmstadt, Germany); commercial ethanol (96%) was acquired from SUQUIN (Bucaramanga, Santander, Colombia).

### 4.2. Vegetal Materials

The *H. colombiana* plants collected in their three stages (vegetative, flowering, and post-flowering) were cultivated in the Pilot Agroindustrial Complex of the National Research Center for Agroindustrialization of Tropical Aromatic and Medicinal Plant Species, CENIVAM, at Universidad Industrial de Santander (Bucaramanga, Santander, Colombia, 07°08.422′ N 073°06.960′ W). The live accessions of wild plants were previously obtained in the botanical outings carried out in the departments of Boyacá and Santander. One of the authors, J.L. Fernández-Alonso (RJB-CSIC), carried out the taxonomic identification of the collected plants of *H. colombiana*, with voucher number 57897, and the control sheet was deposited in the Colombian National Herbarium (COL) of the Institute of Natural Sciences of the National University of Colombia at Bogotá.

**Figure 3:**
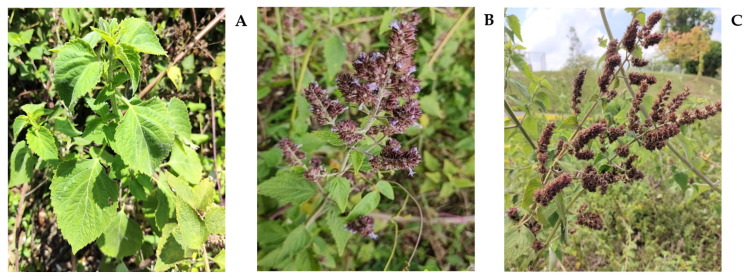
*H. colombiana* plants at three different phenological stages. (**A**) Vegetative, with the new shoots of the perennial plant. (**B**) Flowering, with brownish-purple inflorescences and with lilac flowers. (**C**) Post-flowering with brown spikes; thicker and more compact due to the accrescent calyxes. Photographs taken at CENIVAM-UIS, Bucaramanga (Colombia).

For each experiment, twelve *H. colombiana* plants were collected every two to three months during 2021–2023, according to their phenological stage (vegetative, flowering, and post-flowering). In Figure 3, the plants under study appear at different phenological stages, and in Table S9, the climatic conditions (temperature and humidity) are reported.

### 4.3. Distillation of Essential Oils 

The aerial parts of the *H. colombiana* plants were harvested, dried in a shaded greenhouse, and chopped before their distillation. Plant material (100–350 g) and distilled water (200–350 mL) were added to a round-bottomed flask (2 L) placed inside a household microwave oven (Samsung (Suwon, Republic of Korea), model MS32J5133AG) with 1.6 kW output power and a radiation frequency of 2450 MHz. The total distillation time was 45 min, divided into three periods, each one lasting 15 min, with a 5 min rest time. Hydrodistillation was carried out in Clevenger-type equipment, with a Dean–Stark distillation reservoir with a ground joint to the round-bottomed flask. The EOs obtained were dried with anhydrous Na_2_SO_4_ (J.T. Baker, Phillisburg, NJ, USA) before chromatographic analysis.

### 4.4. Solvent Extraction

Solvent extraction (SE) was performed as described by Medoatinsa et al. [33], with some modifications. To compare the yields and the chemical composition of the extracts, two SEs were carried out using plant materials before or after hydrodistillation, previously dried at room temperature. Dried and ground plant materials of *H. colombiana* (100 g) were mixed with an EtOH:H_2_O solution (2 L, 70:30 *v*/*v*) and deposited in an ultrasonic bath (Elmasonic S15H, Singen, Germany) at 50 °C, for 1 h. The mixture was vacuum filtered using a Büchner funnel (Whatman N° 1 filter paper), a Kitasato flask, and a vacuum pump (Vacuubrand, Wertheim, Germany). The extracts were rotoevaporated in the Heidolph equipment (Hei-VAP, Advantage HL, Chicago, IL, USA), then dried in a VirTis AdVantage Plus tray lyophilizer (SP Scientific, Gardiner, NY, USA) and stored at 4 °C, protected from light, before analysis.

### 4.5. Chromatographic Analysis

#### 4.5.1. GC/MS/FID Essential Oil Analysis

The EOs were analyzed on a GC 6890 Plus Gas Chromatograph (Agilent Technologies, AT, Palo Alto, CA, USA) equipped with an MS 5973 Network 33 Mass Selective Detector (AT, Palo Alto, CA, USA) with electron ionization (EI, 70 eV). Helium (99.995%, AP gas, Messer, Bogotá, Colombia) was used as the carrier gas, with a column head pressure of 113.5 kPa, and a volumetric flow rate of 1 mL/min that was kept constant during all chromatographic runs. Samples were injected in split mode (30:1), and the injector temperature was maintained at 250 °C.

The separation of the EO components was carried out in two capillary columns, one with a polar stationary phase of poly(ethylene glycol) (DB-WAX, J & W Scientific, Folsom, CA, USA) of 60 m × 0.25 mm (i.d.) × 0.25 μm (d_f_) and the other with a nonpolar stationary phase of 5%-phenyl-poly(methylsiloxane), 5%-Ph-PDMS, (DB-5MS, J & W Scientific, Folsom, CA, USA), of the same dimensions as those of the polar column. The temperatures of the ionization chamber and the quadrupole were 230 and 150 °C, respectively. The range of masses was *m*/*z* 45–450 u, with an acquisition speed of 3.58 scan/s. Data were processed with MSDChemStation G1701DA software (AT, Palo Alto, CA, USA). Compound identification was performed based on their linear retention indices (LRIs). The experimental mass spectra of each compound were compared with those of the Adams (2007), NIST (2017), and Wiley (2008) spectral databases.

The quantification of the EO components was performed by an AT 6890N gas chromatograph (AT, Palo Alto, CA, USA), coupled to a flame ionization detector (FID). The separation of the oil components was carried out in a capillary column with the apolar stationary phase (DB-5MS) of the same dimensions as the one used for GC/MS analysis. Samples were injected in split mode (1:30), the injector temperature was 250 °C, the injection volume was 1 μL, and the FID temperature was maintained at 280 °C. Quantification was performed using calibration curves of the reference substances of α-pinene, sabinene, *p*-cymene, limonene, γ-terpinene, linalool, (*E*)-β-caryophyllene, germacrene D, α-humulene, and caryophyllene oxide, among others. The data were processed with GC ChemStation software version B.04.03-SP1 (2001–2012).

#### 4.5.2. LC/MS Extract Analysis

The analysis was carried out in an ultrahigh performance liquid chromatograph, UHPLC Vanquish (Thermo Fisher Scientific, Germering, Germany), equipped with a Q-Exactive Plus Orbitrap mass detector (Thermo Scientific, Bremen, Germany), an electrospray interface with heating (HESI-II), operated in dual acquisition mode of positive and negative ions, at 350 °C, a degasser (SRD-3400), a binary pump (HPG-3400RS), an autosampler (WPS-300TRS), and a thermostated unit (TCC-3000) to house the guard and the analytical columns. Separation of the components of the mixture was performed on a ZORBAX Eclipse XDB-C_18_ column (Sigma Aldrich, St. Louis, MO, USA), 50 mm, L × 2.1 mm, i.d., × 1.8 μm particle size, at 30 °C. The mobile phase was: A—water (0.1% formic acid + 5 mM HCOONH_4_) and B—methanol (0.1% formic acid + 5 mM HCOONH_4_). Nitrogen (>99%) was supplied by an NM32LA generator (Peak Scientific, Inchinnan, Scotland, UK) and used as both the drying and nebulizing gas. The capillary temperature was 320 °C, and the voltage was 3.5 kV. The ions passed through a quadrupole, a C-Trap ion trap, and a higher energy dissociation collision cell (HCD). Values of 10, 20, 30, and 40 eV were used in the HCD. Data were processed with Thermo Xcalibur^TM^ Roadmap software, Version 3.1.66.10. The identification of compounds was carried out based on the extracted ion current (EIC) of protonated [M+H]^+^ or deprotonated [M–H]^−^ molecules, depending on the case, and by comparison with the mass spectra of substances in databases (HMDB [68] and MassBank [69]) and reference substances (Section 4.1).

### 4.6. Evaluation of Antioxidant Activity

#### 4.6.1. Decoloration of the ABTS^+•^ Cation–Radical Assay

The ABTS^+•^ cation–radical decoloration assay was carried out as described by Re et al. [70], with some modifications [71]. The test was performed on a Varioskan LUX VL0000D0 microplate reader (Thermo Scientific, Singapore) using transparent 96-well microplates. ABTS (7 mM in sodium acetate buffer, pH 4.5) and potassium persulfate (2.45 mM) were mixed with sonication for 30 min; the mixture was stored at 4 °C (24 h) in the absence of light to obtain a stable ABTS^+•^ solution. ABTS^+•^ was diluted in acetate buffer until an absorbance of 0.71 ± 0.02 at λ = 750 nm was obtained; the mixture was stored at 4 °C (30 min) before use. The EOs and extracts, obtained from plant materials before and after hydrodistillation, were weighed (10 mg), dissolved in methanol, and diluted in acetate buffer (20 mM, pH 4.5). The EO or the diluted extract and the ABTS^+•^ solution were deposited in each well of the plate and the absorbance was measured for 60 min. Trolox^®^ (97%, Sigma-Aldrich, St. Louis, MO, USA) was used as the reference substance. Three independent experiments were carried out (*n* = 3) and the results were expressed as the mean value ± standard deviation of μmol Trolox^®^/g of EO or dry extract.

#### 4.6.2. Evaluation of the Oxygen Radical Absorption Capacity

The oxygen radical absorbance capacity was determined, according to Ou et al. [72], with some modifications [71]. Measurements were performed using a Varioskan LUX VL0000D0 microplate reader (Thermo Scientific, Singapore) equipped with black 200 μL 96-well poly(styrene) microplates, under fluorescence mode. The EOs or extracts were weighed (1 mg) and dissolved in methanol, and then dilutions were made in phosphate buffer; in each well of the plate, the EO or the diluted extract (25 μL) and the fluorescein solution (150 μL, 81 nM in phosphate buffer) were deposited; the mixture was incubated at 37 °C (20 min), and a solution of AAPH (25 μL, 153 mM, in phosphate buffer) was added. Fluorescence was measured (37 °C, 90 min) with an excitation wavelength of λ = 490 nm and an emission wavelength of λ = 510 nm. The antioxidant protection was determined from the difference between the area under the curve (AUC) obtained for each sample and for the blank (25 μL of phosphate buffer). Trolox^®^ (97%, Sigma-Aldrich, St. Louis, MO, USA) was used as the reference substance. Three replicates were carried out (*n* = 3) and the results were expressed as the mean value ± standard deviation of μmol Trolox^®^/g of EO or dry extract.

### 4.7. Data Analysis

Analysis of variance (ANOVA) was used to determine significant differences (*p* < 0.05) between yields, chemical compositions, and antioxidant activities of *H. colombiana* essential oils and hydroethanolic extracts. ANOVA and Tukey’s test were performed in InfoStat software (Version 2018).

## 5. Conclusions

Thirty-two compounds (relative amount > 0.1%) were found in EOs distilled from *H. colombiana* plants collected at the three phenological stages (vegetative, flowering, and post-flowering); (*E*)-β-caryophyllene, germacrene D, sabinene, and caryophyllene oxide were the most abundant components. Fourteen compounds were identified in the hydroalcoholic extracts obtained from plant material before or after distillation: a pyranone (70 mg/g), rosmarinic acid (17 mg/g), and rutin (8 mg/g) were the major components. The chemical composition of the EOs and extracts of *H. colombiana*, obtained from plants collected at the three phenological stages, did not present appreciable differences in the amounts of their major compounds. The phenological stage of the plant had little effect on the amounts of the main secondary metabolites in the EOs or extracts. The antioxidant activity values obtained from *H. colombiana* extracts showed little variation when the plants were collected in bloom at different harvests. The flowering phenological stage could be considered propitious for the collection of plant materials to obtain extracts with the highest antioxidant activity. The native plant *H. colombiana*, promising for its bioactive compounds, could be easily cultivated in Cundinamarca, Boyacá, Santander, and other regions of Colombia with medium altitudes, where this species has been found growing in poor soils between 600 and 2700 m above sea level [15]. Thus, it could integrate the production chain of natural ingredients for different bioproducts under circular economy schemes that employ the residual biomass from distillation to obtain extracts and individual bioactive compounds of interest to cosmetic and pharmaceutical industries.

## Figures and Tables

**Table 1:** Yields (%) of *H. colombiana* EOs and hydroethanolic extracts.

Phenological Stage	Harvest Date	Total Number of Replicates, *n*	Yield, % (*w*/*w*) ± s		
			Essential Oil	Solvent Extraction	
				Before Distillation	After Distillation
Vegetative	1 October 2021 28 June 2022 12 January 2023	9	0.1 ± 0.1	10 ± 3	6 ± 3
Flowering	30 March 2022 24 November 2022 12 January 2023	9	0.10 ± 0.05	10 ± 3	10 ± 3
Post-flowering	21 January 2022 12 January 2023	6	0.05 ± 0.03	10 ± 2	10 ± 1

**Table 2:** Chemical compositions obtained by GC/MS/FID of the EOs distilled by MWHD from *H. colombiana* plants, collected at three different phenological stages.

N° Figure 1	Compound	Type	Linear Retention Indices				GC/FID Peak Area, % ± s			Identification Criteria
			DB-5MS		DB-WAX					
							Plant Materials Stages			
			LRI_exp_	LRI_lit_	LRI_exp_	LRI_lit_	Vegetative (*n* = 9)	Flowering (*n* = 9)	Post-Flowering (*n* = 6)	
1	α-Thujene	MH	928	928 [35]	1027	1027 [35]	0.3 ± 0.1	0.30 ± 0.07	0.20 ± 0.09	a, b
2	α-Pinene	MH	936	936 [35]	1023	1025 [35]	0.4 ± 0.1	2 ± 1	0.9 ± 0.8	a, b, c
3	Sabinene	MH	976	973 [35]	1124	1122 [35]	10 ± 2	10 ± 3	4 ± 3	a, b, c
4	β-Pinene	MH	981	981 [36]	1111	1110 [35]	2 ± 1	2.0 ± 0.9	1.0 ± 0.9	a, b
5	*p*-Cymene	MH	1028	1024 [35]	1274	1274 [35]	0.5 ± 0.4	0.2 ± 0.1	0.2 ± 0.1	a, b, c
6	Limonene	MH	1032	1030 [36]	1202	1198 [35]	1.0 ± 0.3	1.0 ± 0.2	0.4 ± 0.3	a, b, c
7	(*E*)-β-Ocimene	MH	1047	1044 [34]	1253	1250 [35]	0.30 ± 0.07	1.0 ± 0.7	0.4 ± 0.1	a, b
8	γ-Terpinene	MH	1061	1059 [35]	1249	1245 [35]	0.10 ± 0.05	0.16 ± 0.07	0.10 ± 0.04	a, b, c
9	Linalool	OM	1100	1099 [35]	1550	1549 [37]	0.4 ± 0.3	0.40 ± 0.04	0.4 ± 0.1	a, b, c
10	(*E*)-Sabinene hydrate	OM	1105	1098 [35]	1558	1549 [35]	0.10 ± 0.05	0.20 ± 0.04	0.2 ± 0.1	a, b
11	δ-Elemene	SH	1341	1337 [35]	1473	1469 [36]	1 ± 1	3 ± 3	4 ± 1	a, b
12	α-Cubebene	SH	1351	1448 [34]	1465	1460 [35]	0.20 ± 0.01	0.15 ± 0.02	0.16 ± 0.03	a, b
13	α-Ylangene	SH	1378	1376 [34]	1493	1492 [38]	0.20 ± 0.02	0.20 ± 0.02	0.20 ± 0.03	a, b
14	α-Copaene	SH	1385	1378 [36]	1503	1496 [39]	8 ± 1	6 ± 1	5.0 ± 0.9	a, b
15	β-Bourbonene	SH	1394	1390 [36]	1530	1526 [36]	0.3 ± 0.1	0.40 ± 0.01	1.0 ± 0.3	a, b
16	β-Elemene	SH	1397	1390 [35]	1600	1595 [38]	2 ± 1	3 ± 1	3 ± 1	a, b
17	C_15_H_24_ (Figure S1)	SH	1416	-	1536	-	0.25 ± 0.02	0.20 ± 0.04	0.2 ± 0.1	a, b
18	(*E*)*-*β-Caryophyllene	SH	1435	1428 [36]	1613	1617 [36]	30 ± 4	24 ± 4	30 ± 5	a, b, c
19	γ-Elemene	SH	1438	1436 [35]	1645	1639 [35]	1 ± 1	3 ± 2	2 ± 2	a, b
20	6,9-Guaiadiene	SH	1452	1442 [34]	1617	-	1.5 ± 0.2	1.5 ± 0.1	2.0 ± 0.1	a, b
21	C_15_H_24_ (Figure S2)	SH	1459	-	1630	-	1 ± 1	2 ± 1	2 ± 2	a, b
22	α-Humulene	SH	1469	1465 [36]	1682	1681 [38]	2.0 ± 0.2	2.0 ± 0.2	2.00 ± 0.01	a, b, c
23	γ-Muurolene	SH	1484	1478 [35]	1696	1690 [37]	0.4 ± 0.1	0.5 ± 0.1	0.8 ± 0.1	a, b
24	Germacrene D	SH	1495	1493 [40]	1722	1708 [35]	20 ± 6	20 ± 6	22 ± 1	a, b, c
25	β-Selinene	SH	1499	1490 [34]	1726	1717 [35]	1.0 ± 0.2	1.0 ± 0.2	1.0 ± 0.1	a, b
26	γ-Cadinene	SH	1523	1529 [41]	1767	1766 [36]	2.0 ± 0.6	1.0 ± 0.3	0.80 ± 0.03	a, b
27	δ-Cadinene	SH	1527	1524 [36]	1763	1761 [36]	3.0 ± 0.4	3.0 ± 0.4	2.0 ± 0.5	a, b
28	C_15_H_24_O (Figure S3)	OS	1566	-	1979	-	0.5 ± 0.3	0.4 ± 0.1	0.9 ± 0.1	a, b
29	Germacrene B	OS	1574	1572 [42]	1719	1708 [35]	0.5 ± 0.2	0.7 ± 0.4	1.0 ± 0.6	a, b
30	Caryophyllene oxide	OS	1599	1595 [43]	1996	1993 [44]	9 ± 7	6 ± 2	10 ± 2	a, b, c
31	Viridiflorol	OS	1609	1600 [45]	2093	2089 [35]	0.3 ± 0.2	0.20 ± 0.08	0.3 ± 0.1	a, b
32	C_15_H_24_O (Figure S4)	OS	1640	-	2255	-	2.0 ± 0.3	1 ± 1	2.0 ± 0.2	a, b
Monoterpene hydrocarbons (MH), %							16	18	6	
Oxygenated monoterpenes (OM), %							0.6	0.6	0.6	
Sesquiterpene hydrocarbons (SH), %							70	74	75	
Oxygenated sesquiterpenes (OS), %							12	9	17	

Vegetative stage: plant materials collected 1 October 2021; 28 June 2022; 12 January 2023. Flowering stage: plant materials collected 30 March 2022; 24 November 2022; 12 January 2023. Post-flowering stage: plant materials collected 21 January 2022; 12 January 2023. a. Tentative identification based on linear retention indices (LRIs) determined on the DB-5MS (nonpolar) and DB-WAX (polar) chromatographic columns [34,35,36,37,38,39,40,41,42,43,44,45]. b. Tentative identification based on mass spectra comparison (MS; EI, 70 eV, match > 95%), study of fragmentation patterns, and by comparison of experimental MS with those from Adams (2007), NIST (2017), and Wiley (2008) spectral databases. c. Confirmatory identification based on standard substances (LRIs) (α-pinene (98%, LRI _apolar_: 937, LRI _polar_: 1024), sabinene (95%, LRI _apolar_: 976, LRI _polar_: 1124), p-cymene (99%, LRI _apolar_: 1028, LRI _polar_: 1274), limonene (97%, LRI _apolar_: 1033, LRI _polar_: 1202), γ-terpinene (96%, LRI _apolar_: 1066, LRI _polar_: 1259), linalool (97%, LRI _apolar_: 1102, LRI _polar_: 1551), (*E*)-β-caryophyllene (98.5%, LRI _apolar_: 1434, LRI _polar_: 1611), α-humulene (96%, LRI _apolar_: 1465, LRI _polar_: 1683), germacrene D (95%, LRI _apolar_: 1490, LRI _polar_: 1716), and caryophyllene oxide (95%, LRI _apolar_: 1598, LRI _polar_: 1996)), and the comparison of their mass spectra and retention times (t_R_) with those of the compounds present in the EOs studied.

**Table 3:** Antioxidant activity of the EOs, hydroethanolic extracts, and specific terpene and phenolic compounds found in *H. colombiana* EOs and extracts.

Phenological Stages	*n* *	μmol Trolox^®^/g Sample, ± s					
		ABTS^+•^			ORAC		
		Essential Oil	Extract *		Essential Oil	Extract *	
			Before Distillation	After Distillation		Before Distillation	After Distillation
Vegetative	9	292 ± 106	1990 ± 144	2260 ± 440	610 ± 69	4644 ± 1142	5393 ± 1992
Flowering	9	390 ± 114	1780 ± 191	1940 ± 97	632 ± 11	4670 ± 514	4274 ± 1047
Post-flowering	6	904 ± 2	2274 ± 83	2180 ± 255	540 ± 42	7690 ± 4345	4950 ± 1260
Standard Compounds	*n*	ABTS^+•^			ORAC		
Rosmarinic acid	3		3260 ± 116			32030 ± 327	
Caffeic acid			4510 ± 92			23400 ± 80	
Rutin			1190 ± 37			8210 ± 11	
Sabinene			25.0 ± 0.5			350 ± 13	
(*E*)-β-Caryophyllene			**			440 ± 27	
Caryophyllene oxide			**			540 ± 34	

* Total of number replicates, *n*. ** No antioxidant activity registered at 2.00 g/L.

## Data Availability

The supporting data are found in the database of the CIBIMOL research group, Universidad Industrial de Santander, Bucaramanga, Colombia.

## Supplementary Materials

The following supporting information can be downloaded online: https://www.mdpi.com/article/10.3390/molecules28196767/s1, Table S1. Results of the analysis of variance, which was conducted to assess how different phenological stages of *H. colombiana* plants affect the yields of EO and plant material extracts before and after distillation. Table S2. Calibration equations employed for *H. colombiana* EO and extract components quantification. Table S3. Quantification by GC/FID of some EO components of *H. colombiana* collected at three phenological stages. Table S4. Results of the analysis of variance, which were used to evaluate the impact of plant material at various phenological stages on the chemical composition of the EO distilled by MWHD from *H. colombiana* plants. Table S5. Exact masses of deprotonated [M–H]^−^ and protonated [M+H]^+^ molecules, identified by UHPLC-ESI^+/−^-Orbitrap-MS, of the compounds in the hydroethanolic extracts obtained from *H. colombiana* before and after its distillation. Table S6. Quantification of some compounds present in the extracts of *H. colombiana*, obtained from plants collected at three different phenological stages, before and after their distillation. Table S7. Results of the analysis of variance, which was conducted to assess how different phenological stages of *H. colombiana* plants affect the chemical composition of plant material extracts before and after distillation. Table S8. Results of the analysis of variance used to evaluate the effect of the phenological state of the plant material on the antioxidant activities of EOs and hydroethanolic extracts of the *H. colombiana* before and after their distillation. Table S9. Registered environmental conditions during the days of plant harvest. Figure S1. Mass spectrum of sesquiterpene C_15_H_24_, peak N° 17 (Figure 1). Nonpolar column LRI: 1416; polar column LRI: 1536 (Table 2), [M^+•^] *m*/*z* 204 (100%); [M–CH_3_]^+^ *m*/*z* 189 (68%); [M–C_2_H_5_]^+^ *m*/*z* 175 (12%); [M–C_3_H_7_]^+^ *m*/*z* 161 (28%). Figure S2. Mass spectrum of sesquiterpene C_15_H_24_, peak N° 21 (Figure 1). Nonpolar column LRI: 1459; polar column LRI: 1630 (Table 2) [M^+•^] *m*/*z* 204 (60%); [M–CH_3_]^+^ *m*/*z* 189 (35%); [M–C_2_H_5_]^+^ *m*/*z* 175 (5%); [M–C_3_H_7_]^+^ *m*/*z* 161 (100%), C_3_H_5_^+^ *m*/*z* 41 (23%). Figure S3. Mass spectrum of oxygenated sesquiterpene C_15_H_24_O, peak N° 28 (Figure 1). Nonpolar column LRI: 1566; polar column LRI: 1979 (Table 2) [M^+•^] *m*/*z* 220 (2%); [M–CH_3_]^+^ *m*/*z* 205 (12%); [M–H_2_O]^+•^ *m*/*z* 202 (7%); [M–C_2_H_5_]^+^ *m*/*z* 191 (10%), CH_3_CO^+^ *m*/*z* 43 (53%); C_3_H_5_^+^ *m*/*z* 41 (55%). Figure S4. Mass spectrum of unidentified compound, C_15_H_24_O, peak N° 32 (Figure 1). Nonpolar column LRI: 1640; polar column LRI: 2255 (Table 2). [M^+•^] *m*/*z* 220 (10%); [M–CH_3_]^+^ *m*/*z* 205 (25%), [M–H_2_O]^+•^ *m*/*z* 202 (25%), [M–CH_3_–H_2_O]^+^ *m*/*z* 187 (28%). Figure S5. Extracted ion currents from protonated [M+H]^+^ or deprotonated [M–H]^−^ molecules, obtained by UHPLC-ESI^+/−^-Orbitrap-MS (scale 6.2 × 10^7^), of compounds in the hydroalcoholic extracts of *H. colombiana*, isolated from post-distillation residues of plants collected at different phenological stages. (A) Vegetative stage. (B) Flowering stage. (C) Post-flowering stage. Compound identification appears in Table S5. Figure S6. Mass spectrum of the protonated [M+H]^+^ molecule of a pyranone, at *m*/*z* 427.16000, obtained using the selected ion monitoring mode (SIM) with HCD 10 eV.

## Sample Availability

Samples of *H. colombiana* EOs and extracts are available at CROM-MASS—Universidad Industrial de Santander (Bucaramanga, Colombia).

## Abbreviations

ABTS	2,2-Azinobis-(3-ethylbenzothiazoline-6-sulfonic acid)-diammonium salt assay
AD	After distillation
AT	Agilent Technologies
BD	Before distillation
d_f_	Film thickness
i.d.	Internal Diameter
EIC	Extracted Ion Chromatogram
EO(s)	Essential oil(s)
ESI	Electrospray ionization
eV	Electron volt
FID	Flame ionization detection
GC	Gas chromatography or gas chromatogram
HCD	Higher-Energy Collision Dissociation Cell
HRMS	High-Resolution Mass Spectrometry
I, %	Intensity (Abundance)
LC	Liquid Chromatography
LOD	Limit of Detection
LOQ	Limit of Quantification
LRI	Linear Retention Index
MS	Mass Spectrometry or Mass Spectrum
MSD	Mass Spectrometric Detector
*m*/*z*	Mass-to-Charge Ratio
MWHD	Microwave-Assisted Hydrodistillation
ORAC	Oxygen Radical Absorption Capacity Assay
t_R_	Retention time (min)
UHPLC	Ultrahigh Performance Liquid Chromatography
