# Supplementary material for: Antioxidant Properties and Secondary Metabolites Profile of Hyptis colombiana at Various Phenological Stages
Source: Molecules. 2023 Sep 22;28(19):6767. doi: 10.3390/molecules28196767 (PMC10574317; doi:10.3390/molecules28196767)
Supplement: Supplementary file 1 [file molecules-28-06767-s001.zip › molecules-2522853-supplementary.pdf]

molecules

MDPI

Supplementary Materials

Antioxidant Properties and Secondary Metabolites Profile of *Hyptis colombiana* at Various Phenological Stages

Sheila B. Beltrán [1], Lady J. Sierra [1], José L. Fernández-Alonso [2], Angie K. Romero [1], Jairo R. Martínez [1] and Elena E. Stashenko [1,*]

[1] Centro de Investigación en Biomoléculas-CIBIMOL, Laboratorio de Cromatografía y Espectrometría de Masas-CROM-MASS, Universidad Industrial de Santander, Bucaramanga 680002, Colombia; sheila2218097@correo.uis.edu.co (S.B.B.); lady.sierra2@correo.uis.edu.co (L.J.S.); cenivam.sgr3@uis.edu.co (A.K.R.); jmartine@uis.edu.co (J.R.M.)
[2] Real Jardín Botánico—CSIC, Claudio Moyano 1, 28014 Madrid, Spain; jlfernandeza@rjb.csic.es
* Correspondence: elena@tucan.uis.edu.co

Table S1. Results of the analysis of variance, which were conducted to assess how different phenological stages of *H. colombiana* plants affect the yields of EO and plant material extracts before and after distillation.

Sample	Yield				
	Vegetative	Flowering	Post-Flowering	F	*p*
EO	A	A	A	1.10	0.4171
Extract before distillation	A	A	A	0.38	0.7026
Extract after distillation	A	A	A	1.13	0.4079

Different letters in the column indicate significant differences. A p value < 0.05 indicates that the effect is significant with a 95% confidence level. F-value.

Table S2. Calibration equations employed for *H. colombiana* EO and extract components quantification.

Compounds	Method	Linear Equations [a]	R^2	mg/kg	
				LOD	LOQ
α-Pinene	GC/FID	y = 0.518654905x + 0.632455296	0.9992	10	30
Sabinene		y = 0.649032603x – 6.278687321	0.9923	45	150
p-Cymene		y = 0.655622219x + 11.11241102	0.9839	80	260
Limonene		y = 0.615370539x + 0.858374656	0.9954	5	20
γ-Terpinene		y = 0.598137888x + 2.102415535	0.9959	5	20
Linalool		y = 0.615370539x + 0.858374656	0.9955	5	20
(*E*)-β-Caryophyllene		y = 0.867898976x – 10.0313885	0.9965	10	20
α-Humulene		y = 0.625073888x + 35.53629045	0.9956	5	10
Caryophyllene oxide		y = 0.936781157x – 99.66879431	0.9952	20	50
p-Hydroxybenzoic acid	LC/MS	y = 1870798x + 81740	0.9976	0.05	0.17
Caffeic acid		y = 18668078x + 29291	0.9986	0.03	0.12
Vitexin		y = 21780885x + 192646	0.9982	0.04	0.15
o-Hydroxybenzoic acid		y = 19492231x + 517415	0.9987	0.03	0.11
Luteolin-7-*O*-glucoside		y = 5837043x + 108532	0.9909	0.09	0.30
Rutin		y = 1530405x + 277504	0.9922	0.09	0.33
Rosmarinic acid		y = 19333680x – 9756668	0.9927	0.08	0.30
Kaempferol-3-*O*-rutinoside		y = 2393025x + 6055	0.9968	0.06	0.20
Salvigenin		y = 534858152x – 2321806	0.9968	0.05	0.15

[a] Calibration curves obtained by the external standard method. LOQ: Limit of quantitation. LOD: Limit of detection.

Table S3. Quantification by GC/FID of some EO components of *H. colombiana* collected at three phenological stages.

Compound	Formula	Type	mg/g EO ± s		
			Plant Material Stage		
			Vegetative (*n* = 9)	Flowering (*n* = 9)	Post-Flowering (*n* = 6)
α-Pinene	$C_{10}H_{16}$	MH	5 ± 1	30 ± 18	10 ± 10
Sabinene	$C_{10}H_{16}$	MH	150 ± 9	140 ± 19	40 ± 40
β-Pinene[1]	$C_{10}H_{16}$	MH	30 ± 13	30 ± 7	10 ± 10
p-Cymene	$C_{10}H_{14}$	MH	<LOD	<LOD	<LOD
Limonene	$C_{10}H_{16}$	MH	10 ± 2	10 ± 2	4 ± 4
γ-Terpinene	$C_{10}H_{16}$	MH	<LOQ	2.0 ± 0.7	<LOQ
Linalool	$C_{10}H_{18}O$	OM	5 ± 3	4 ± 1	4 ± 1
δ-Elemene[2]	$C_{15}H_{24}$	SH	<LOQ	38 ± 32	30 ± 18
α-Copaene [2]	$C_{15}H_{24}$	SH	75.0 ± 0.9	60 ± 6	50 ± 8
β-Elemene[2]	$C_{15}H_{24}$	SH	30 ± 16	30 ± 14	30 ± 11
(*E*)-β-Caryophyllene	$C_{15}H_{24}$	SH	230 ± 74	230 ± 37	250 ± 11
γ-Elemene[2]	$C_{15}H_{24}$	SH	15 ± 15	29 ± 24	30 ± 22
6,9-Guaiadiene[2]	$C_{15}H_{24}$	SH	10 ± 4	15 ± 2	20.5 ± 0.1
α-Humulene	$C_{15}H_{24}$	SH	20 ± 6	20 ± 5	17.70 ± 0.06
Germacrene D[2]	$C_{15}H_{24}$	SH	150 ± 77	210 ± 89	210 ± 16
δ-Cadinene[2]	$C_{15}H_{24}$	SH	30 ± 7	25 ± 1	20 ± 4
Caryophyllene oxide	$C_{15}H_{24}O$	OS	80 ± 46	60 ± 10	130 ± 31

MH: Monoterpene hydrocarbons, OM: Oxygenated monoterpenes, SH: Sesquiterpen hydrocarbons, OS: Oxygenated sesquiterpenes. LOQ: Limit of quantification. LOD: Limit of detection. 1: Amount expressed as α-pinene equivalents. 2: Amount expressed as (*E*)-β-caryophyllene. Equivalents.

Table S4. Results of the analysis of variance, which were used to evaluate the impact of plant material at various phenological stages on the chemical composition of the EO distilled by MWHD from *H. colombiana* plants.

Compounds	mg/g EO				
	Vegetative	Flowering	Post-Flowering	F	*p*
α-Pinene	A	A	A	2.48	0.1997
Sabinene	B	B	A	11.24	0.0228
(*E*)-β-Caryophyllene	A	A	A	0.15	0.8625
Germacrene D	A	A	A	0.55	0.6175
Caryophyllene oxide	A	A	A	3.17	0.1494

Different letters in the column indicate significant differences. A p value < 0.05 indicates that the effect is significant with a 95% confidence level. F-value.

Table S5. Exact masses of deprotonated $[M–H]^–$ and protonated $[M+H]^+$ molecules, identified by UHPLC-ESI$^{+/-}$-Orbitrap-MS, of the compounds in hydroethanolic extracts obtained from *H. colombiana* before and after its distillation.

N° Figs. 2 y S5	Compound	Formula	Exp. Masses *m/z* (I, %)	Δppm	HCD, eV	Product-Ions	Formula	*m/z*, I (%)	Identification Criteria	Reference
1	*p*-Hydroxybenzoic acid isomer	$C_7H_6O_3$	$[M–H]^–$ 137.02330 (100)	0.01	20	$[(M–H)–CO_2]^–$	C_6H_5O	93.03345 (15)	a	
2	Caffeic acid	$C_9H_8O_4$	$[M–H]^–$ 179.03401 (22)	3.02	10	$[(M–H)–CO_2]^–$	$C_8H_7O_2$	135.04446 (100)	a, b, c	[25]
3	Apigenin-*C,C*-dihexoside	$C_{27}H_{30}O_{15}$	$[M+H]^+$ 595.16498 (100)	1.29	10	$[(M+H)–H_2O]^+$	$C_{27}H_{29}O_{14}$	577.14665 (39)	a	
						$[(M+H)–2H_2O]^+$	$C_{27}H_{27}O_{13}$	559.14392 (7)		
						$[(M+H)–3H_2O]^+$	$C_{27}H_{25}O_{12}$	541.13464 (2)		
						$[(M+H)–2H_2O–C_4H_6O_3]^+$	$C_{23}H_{21}O_{10}$	457.12061 (10)		
						$[(M+H)–3H_2O– C_6H_{10}O_5]^+$	$C_{21}H_{15}O_7$	379.08102 (1)		
4	*o*-Hydroxybenzoic acid	$C_7H_6O_3$	$[M–H]^–$ 137.02335 (36)	0.02	20	$[(M–H)–CO_2]^–$	C_6H_5O	93.03345 (100)	a	
5	Luteolin-*C*-hexoside-*O*-desoxyhexosyl	$C_{27}H_{30}O_{15}$	$[M+H]^+$ 595.16473 (100)	1.7	10	$[(M+H)–H_2O]^+$	$C_{27}H_{29}O_{14}$	577.15497 (14)	a	
						$[(M+H)–2H_2O]^+$	$C_{27}H_{27}O_{13}$	559.14545 (5)		
						$[(M+H)–C_6H_{10}O_5]^+$	$C_{21}H_{21}O_{10}$	433.11267 (11)		
6	Luteolin-7-*O*-glucoside	$C_{21}H_{20}O_{11}$	$[M+H]^+$ 449.10681 (0.5)	1.13	20	$[(M+H)–C_6H_{10}O_5]^–$	$C_{15}H_{11}O_6$	287.05472 (100)	a, c	
7	Rutin	$C_{27}H_{30}O_{16}$	$[M+H]^+$ 611.15903 (1)	1.63	10	$[(M+H)–C_6H_{10}O_4]^+$	$C_{21}H_{21}O_{12}$	465.09952 (1)	a, b, c	[25]
						$[(M+H)–C_6H_{10}O_4–C_6H_{10}O_5]^+$	$C_{15}H_{11}O_7$	303.04858 (100)		
8	Rosmarinic acid	$C_{18}H_{16}O_8$	$[M–H]^–$ 359.07642 (37)	0.27	10	$[(M–H)–C_9H_6O_3]^–$	$C_9H_9O_5$	197.04992 (32)	a, b, c	[24,26]
						$[(M–H)–C_9H_{10}O_5]^–$	$C_9H_5O_3$	161.02354 (100)		
9	Kaempferol-3-*O*-rutinoside	$C_{27}H_{30}O_{15}$	$[M+H]^+$ 595.16486 (11)	1.49	10	$[(M+H)–C_6H_{10}O_4]^+$	$C_{21}H_{21}O_{11}$	449.10736 (36)	a, c	
						$[(M+H)–C_6H_{10}O_4–C_6H_{10}O_5]^+$	$C_{15}H_{11}O_6$	287.05469 (100)		
10	Pyranone (**Figure S6**)	$C_{20}H_{26}O_{10}$	$[M+H]^+$ 427.16000 (0.1)	0.30	10	$[(M+H)–C_2H_4O_2]^+$	$C_{18}H_{23}O_8$	367.13846 (100)	a, b	[46]
						$[(M+H)–C_2H_4O_2–C_2H_2O]^+$	$C_{16}H_{21}O_7$	325.12781 (30)		
						$[(M+H)–C_2H_4O_2–C_2H_2O–C_2H_4O_2]^+$	$C_{14}H_{17}O_5$	265.10681(41)		
						$[(M+H)–C_2H_4O_2–C_2H_2O–C_2H_4O_2 –H_2O]^+$	$C_{14}H_{15}O_4$	247.09605 (7)		
						$[(M+H)–C_2H_4O_2–C_2H_2O–C_2H_4O_2–C_2H_2O]^+$	$C_{12}H_{15}O_4$	223.09624 (3)		

Table S5. Continuation.

11	Sesquiterpene lactone	$C_{15}H_{22}O_3$	$[M+H]^+$ 251.16396 (52)	0.85	30	$[(M+H)–H_2O]^+$	$C_{15}H_{21}O_2$	233.15338 (4)		
						$[(M+H)–2H_2O]^+$	$C_{15}H_{19}O$	215.14297 (1.2)		
						$[(M+H)–H_2O–CO]^+$	$C_{14}H_{21}O$	205.15865 (43)		
						$[(M+H)–H_2O–CO–H_2O]^+$	$C_{14}H_{19}$	187.14807 (8)		
12	Hydroxylated salvigenin	$C_{18}H_{16}O_7$	$[M+H]^+$ 345.09641(22)	0.47	40	$[(M+H)–CH_3]^{+•}$	$C_{17}H_{14}O_7$	330.02791(22)	a, b	[27]
						$[(M+H)–2CH_3]^+$	$C_{16}H_{11}O_7$	315.04947 (100)		
						$[(M+H)–2CH_3–CO]^+$	$C_{15}H_{11}O_6$	287.05432 (2.7)		
13	Trihydroxy–trimethoxyflavone	$C_{18}H_{16}O_8$	$[M+H]^+$ 361.09131 (27)	1.34	40	$[(M+H)–CH_3]^{+•}$	$C_{17}H_{14}O_8$	346.06790 (28)	a	
						$[(M+H)–2CH_3]^+$	$C_{16}H_{11}O_8$	331.04444 (100)		
						$[(M+H)–CH_3-H_2O]^{+•}$	$C_{17}H_{12}O_7$	328.05746 (30)		
						$[(M+H)–2CH_3-CO]^+$	$C_{15}H_{11}O_7$	303.04956 (56)		
14	Salvigenin	$C_{18}H_{16}O_6$	$[M+H]^+$ 329.10156 (63)	0.40	40	$[(M+H)–CH_3]^{+•}$	$C_{17}H_{14}O_6$	314.07813 (33)	a, c	
						$[(M+H)–CH_3–H_2O]^{+•}$	$C_{17}H_{12}O_5$	296.06760 (100)		
						$[(M+H)–CH_3–H_2O–CO]^{+•}$	$C_{16}H_{12}O_4$	268.07272 (56)		
						$[(M+H)–CH_3–H_2O–2CO]^{+•}$	$C_{15}H_{12}O_3$	240.07790 (2)		

[a] Identification based on exact mass measurement, isotopic ion intensity ratios, and the study of fragmentation patterns of protonated and deprotonated molecules, and their comparison with mass spectra of the *HMDB* 4.0 [68] and *Massbank* [69] databases. [b] Identification based on scientific literature data on secondary metabolites of species of genus *Hyptis* spp. [24-27, 46]. [c] Confirmatory identification based on the comparison of mass spectra and experimental retention times (t_R) with those of standard substances obtained from *Sigma Aldrich*, *p*-hydroxybenzoic (99%), caffeic acid (98%), *o*-hydroxybenzoic (99%), rosmarinic acid (97%), rutin (94%), salvigenin (98%), kaempferol-3-rutinoside (97%) y luteolin-7-glucoside (98%).

Table S6. Quantification of some compounds present in the extracts of *H. colombiana*, obtained from plants collected at different phenological stages, before and after their distillation

Compound	mg/g extract ± s					
	Vegetative Stage (n = 9)		Flowering Stage (n = 9)		Post-flowering Stage (n = 6)	
	BD	AD	BD	AD	BD	AD
p-Hydroxybenzoic acid isomer[1]	8 ± 6	16 ± 2	4.0 ± 0.8	10 ± 2	4.0 ± 0.7	12.0 ± 0.4
Caffeic acid	1.0 ± 0.3	1.40 ± 0.06	0.60 ± 0.06	1.0 ± 0.3	0.80 ± 0.09	1.100 ± 0.003
Apigenin-*C*,*C*-diglucoside[2]	0.40 ± 0.04	0.250 ± 0.001	0.2 ± 0.1	0.2 ± 0.1	0.20 ± 0.04	0.20 ± 0.03
o-Hydroxybenzoic acid	<LOD	<LOQ	<LOD	<LOQ	<LOQ	0.430 ± 0.009
Luteolin-*C*-hexoside-*O*-desoxyhexosyl[2]	<LOD	<LOD	<LOD	<LOD	<LOD	<LOD
Luteolin-7-*O*-glucoside	1.60 ± 0.07	<LOQ	2 ± 1	2 ± 1	1.70 ± 0.05	1.5 ± 0.2
Rutin	10 ± 4	8 ± 1	10 ± 2	6 ± 2	10 ± 5	8.0 ± 0.6
Rosmarinic acid	20 ± 4	20 ± 8	14 ± 3	20 ± 8	20 ± 8	18 ± 1
Kaempferol-3-rutinoside	0.6 ± 0.3	0.345 ± 0.002	0.5 ± 0.1	0.30 ± 0.09	0.7 ± 0.5	0.50 ± 0.06
Pyranone[3]	70 ± 44	40 ± 1	60 ± 33	40 ± 12	80 ± 17	70 ± 5
Sesquiterpene lactone[4]	<LOD	<LOQ	<LOD	<LOD	<LOD	<LOD
Hydroxylated salvigenin[4]	0.6 ± 0.2	0.86 ± 0.03	0.40 ± 0.06	0.2 ± 0.1	0.20 ± 0.02	0.140 ± 0.005
Trihydroxy-trimethoxyflavone[4]	0.125 ± 0.002	0.136 ± 0.001	<LOQ	0.14 ± 0.01	<LOQ	<LOQ
Salvigenin	0.25 ± 0.04	0.5 ± 0.1	0.20 ± 0.08	0.20 ± 0.07	<LOQ	<LOQ

a Calibration curves obtained with the external standard method. Vegetative stage: plant material collected 1 October 2021; 28 June 2022; 12 January 2023. Flowering stage: plant material collected 30 March 2022; 24 November 2022; 12 January 2023. Post-flowering stage: plant material collected 21 January 2022; 12 January 2023. BD: Before distillation; AF: After distillation. 1: Amount expressed as *p*-hydroxybenzoic acid equivalents. 2: Amount expressed as vitexin equivalents. 3: Amount expressed as rutin equivalents. 4: Amount expressed as salvigenin equivalents. LOD: Limit of detection. LOQ: Limit of quantification.

Table S7. Results of the analysis of variance, which were conducted to assess how different phenological stages of *H. colombiana* plants affect the chemical composition of plant material extracts before and after distillation.

Compound	mg Compound/g Extract									
	Extract before Distillation					Extract after Distillation				
	Vegetative	Flowering	Post-Flowering	F	*p*	Vegetative	Flowering	Post-Flowering	F	*p*
p-Hydroxybenzoic acid isomer	A	A	A	1.04	0.4191	A	A	A	4.25	0.1023
Rutin	A	A	A	0.63	0.5709	A	A	A	0.42	0.6832
Rosmarinic acid	A	A	A	0.95	0.4473	A	A	A	1.6×10^{-3}	0.9984
Pyranone	A	A	A	0.20	0.8223	A	A	A	7.86	0.0411

Different letters in the column indicate significant differences. A *p* value < 0.05 indicates that the effect is significant with a 95% confidence level. F-value.

Table S8. Results of the analysis of variance used to evaluate the effect of the phenological state of the plant material, on the antioxidant activities of EOs and hydroethanolic extracts of the *H. colombiana*, before and after their distillation.

Assay	Sample	Antioxidant Activities				
		Vegetative	Flowering	Post-Flowering	F	*p*
ABTS$^{+\bullet}$	EO	A	A	B	38.85	0.0009
	Extract before distillation	A,B	A	B	6.07	0.0460
	Extract after distillation	A	A	A	1.23	0.3845
ORAC	EO	A	A	A	3.19	0.1139
	Extract before distillation	A	A	A	1.57	0.2964
	Extract after distillation	A	A	A	0.41	0.6907

Different letters in the column indicate significant differences. A *p* value < 0.05 indicates that the effect is significant with a 95% confidence level. F-value.

Table S9. Registered environmental conditions during the days of plant harvest.

Plant Development Stages	Collection Date	Total of Number Replicates, *n*	Environmental Conditions	
			Mean Ambient Temperature, °C	Mean Ambient Humidity, %
Vegetative	1 October 2021<br>28 June 2022<br>12 January 2023	9	30 ± 3	70 ± 23
Flowering	30 March 2022<br>24 November 2022<br>12 January 2023	9	30 ± 2	50 ± 28
Post-flowering	21 January 2022<br>12 January 2023	6	30 ± 2	60 ± 19

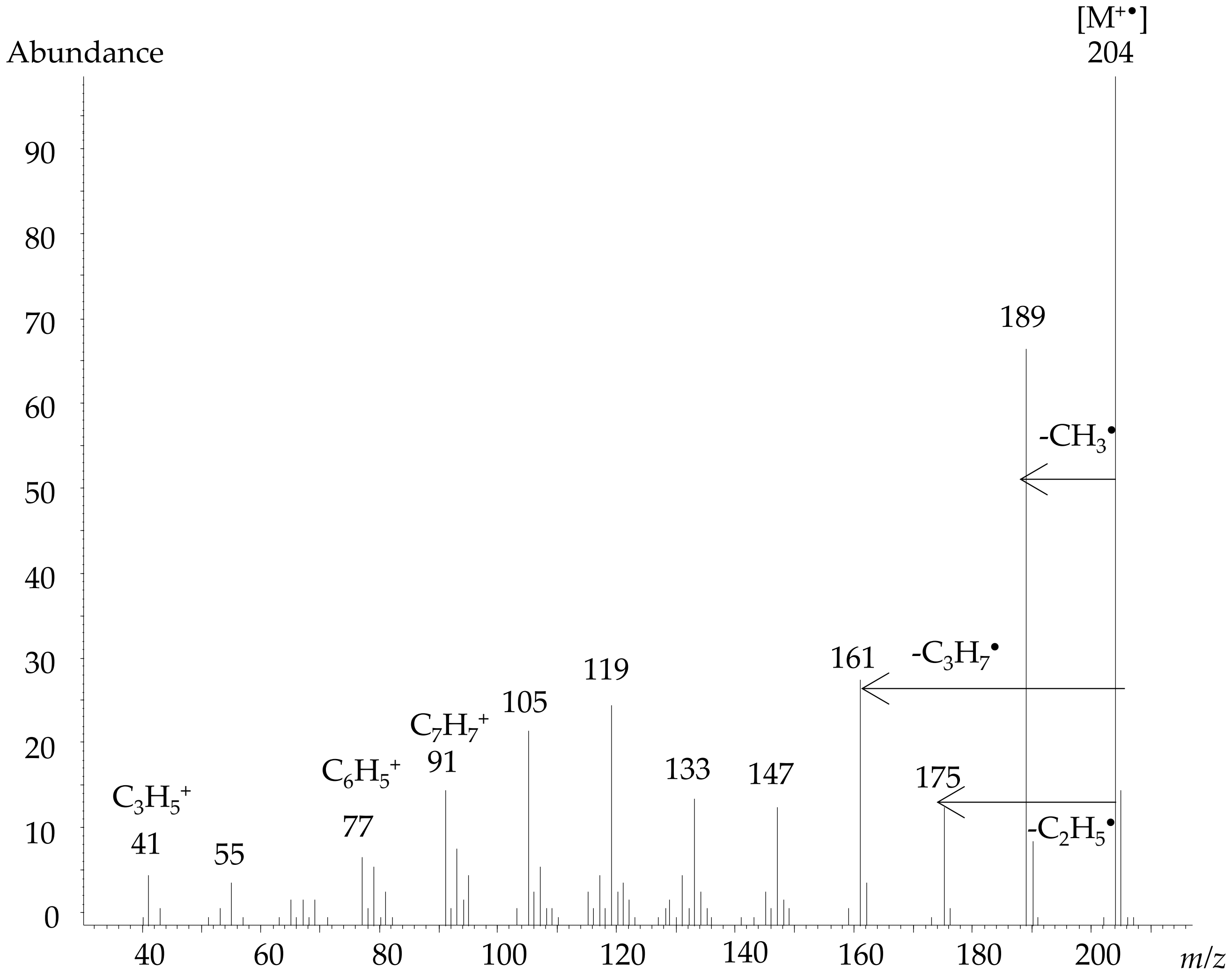

Figure S1. Mass spectrum of sesquiterpene $C_{15}H_{24}$, peak N° 17 (Figure 1). Nonpolar column LRI: 1416; polar column LRI: 1536 (Table 2), $[M^{+\bullet}]$ *m*/*z* 204 (100%); $[M{-}CH_3]^+$ *m*/*z* 189 (68%); $[M{-}C_2H_5]^+$ *m*/*z* 175 (12%); $[M{-}C_3H_7]^+$ *m*/*z* 161 (28%).

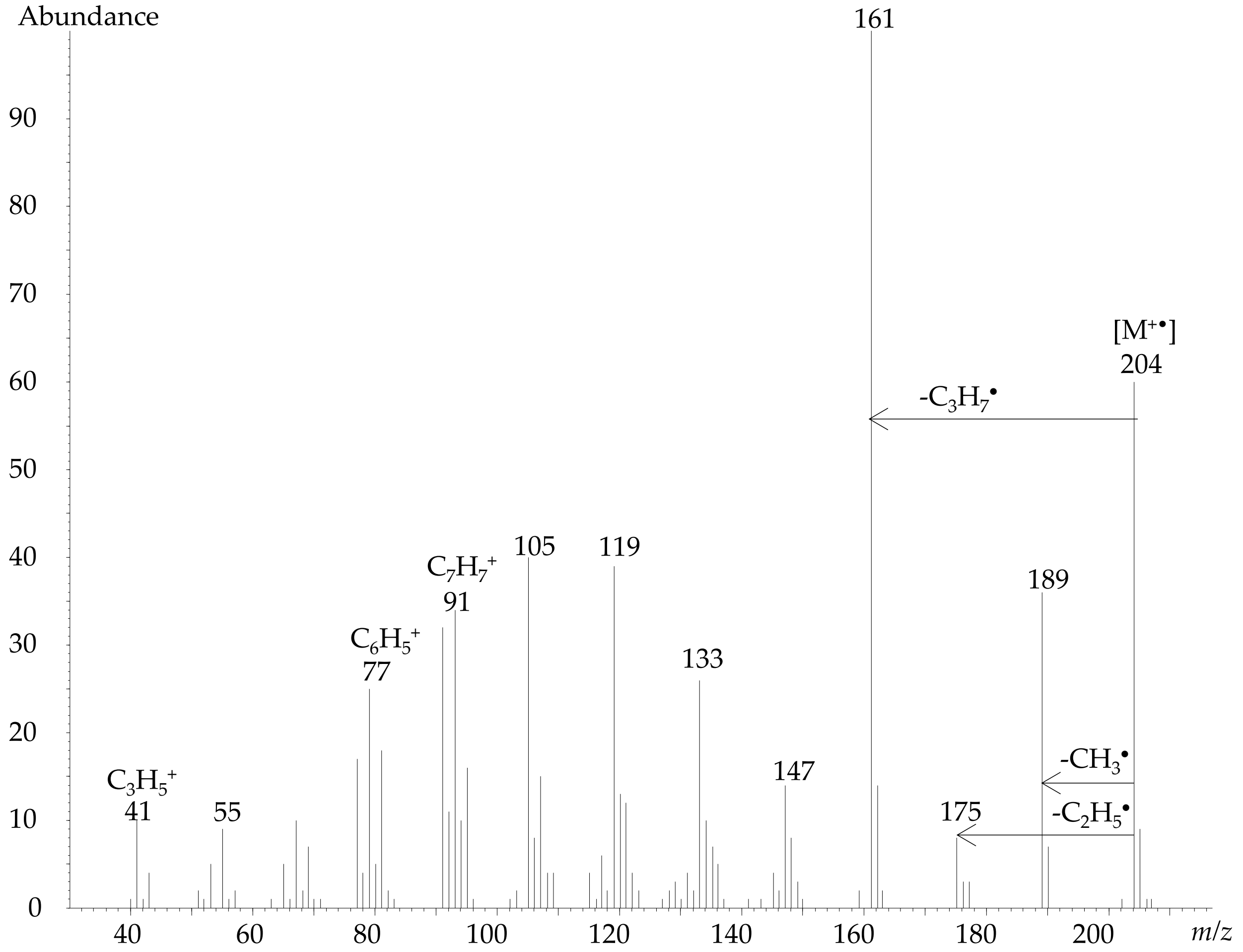

Figure S2. Mass spectrum of sesquiterpene $C_{15}H_{24}$, peak N° 21 (Figure 1). Nonpolar column LRI: 1459; polar column LRI: 1630 (Table 2) $[M^{+\bullet}]$ *m/z* 204 (60%); $[M–CH_3]^+$ *m/z* 189 (35%); $[M–C_2H_5]^+$ *m/z* 175 (5%); $[M–C_3H_7]^+$ *m/z* 161 (100%), $C_3H_5^+$ *m/z* 41 (23%).

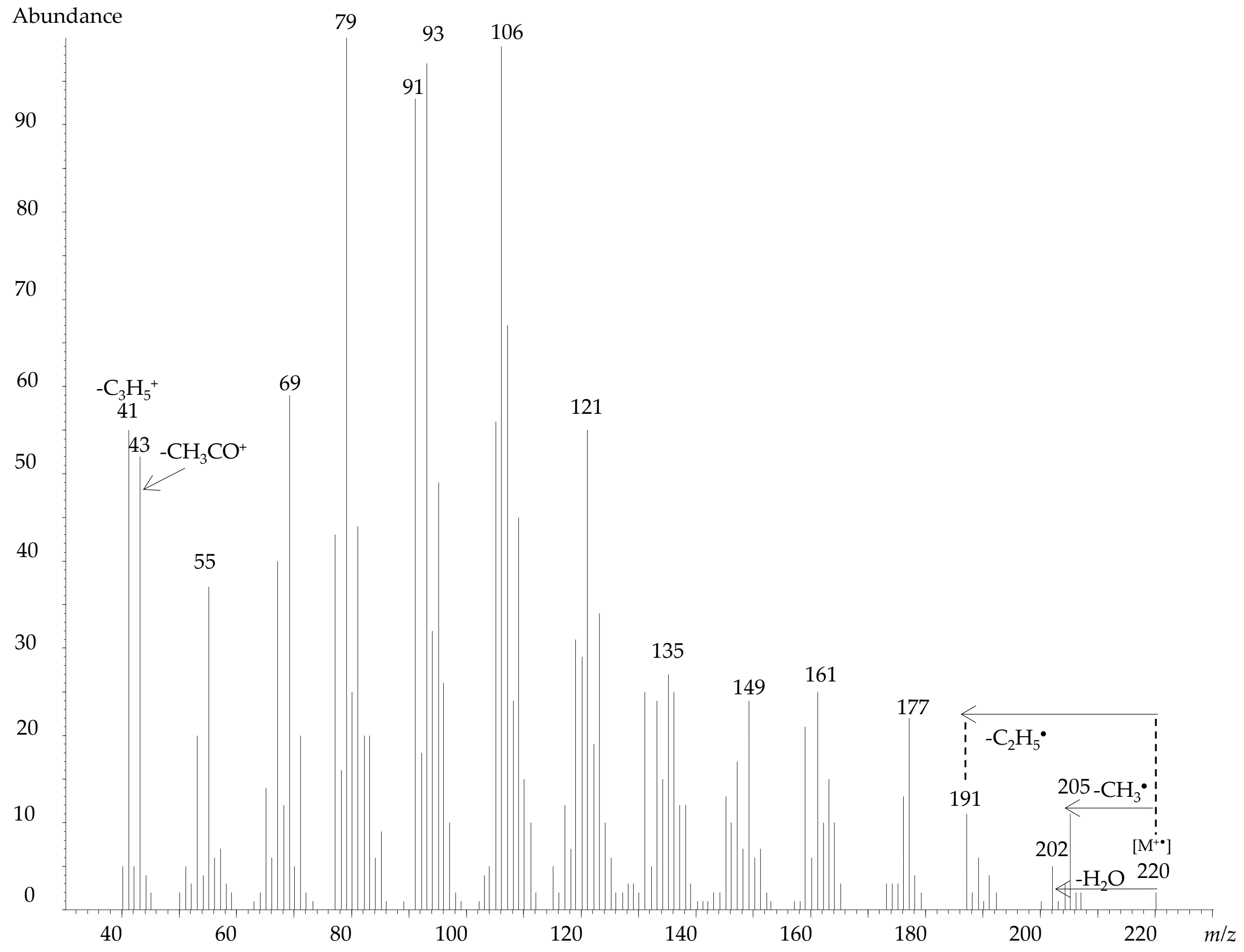

Figure S3. Mass spectrum of oxygenated sesquiterpene $C_{15}H_{24}O$, peak N° 28 (Figure 1). Nonpolar column LRI: 1566; polar column LRI: 1979 (Table 2) $[M^{+\bullet}]$ *m/z* 220 (2%); $[M–CH_3]^+$ *m/z* 205 (12%); $[M–H_2O]^{+\bullet}$ *m/z* 202 (7%); $[M–C_2H_5]^+$ *m/z* 191 (10%), CH_3CO^+ *m/z* 43 (53%); $C_3H_5^+$ *m/z* 41 (55%).

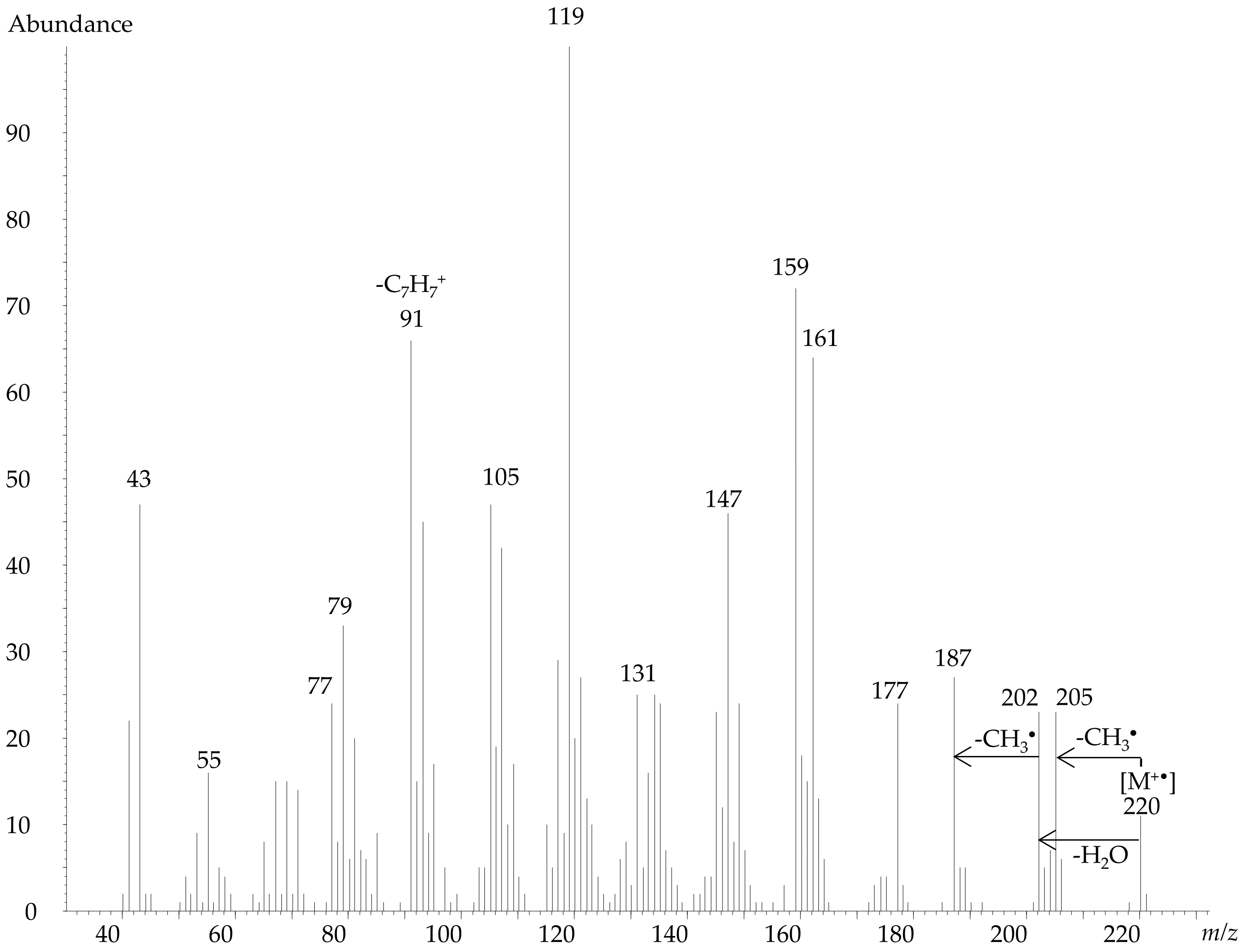

Figure S4. Mass spectrum of oxygenated sesquiterpene $C_{15}H_{24}O$, peak N° 32 (Figure 1). Nonpolar column LRI: 1640; polar column LRI: 2255 (Tabla 2) [$M^{+\bullet}$] *m/z* 220 (10%); [M–CH_3]$^+$ *m/z* 205 (25%), [M–H_2O]$^{+\bullet}$ *m/z* 202 (25%), [M–CH_3–H_2O]$^+$ *m/z* 187 (28%).

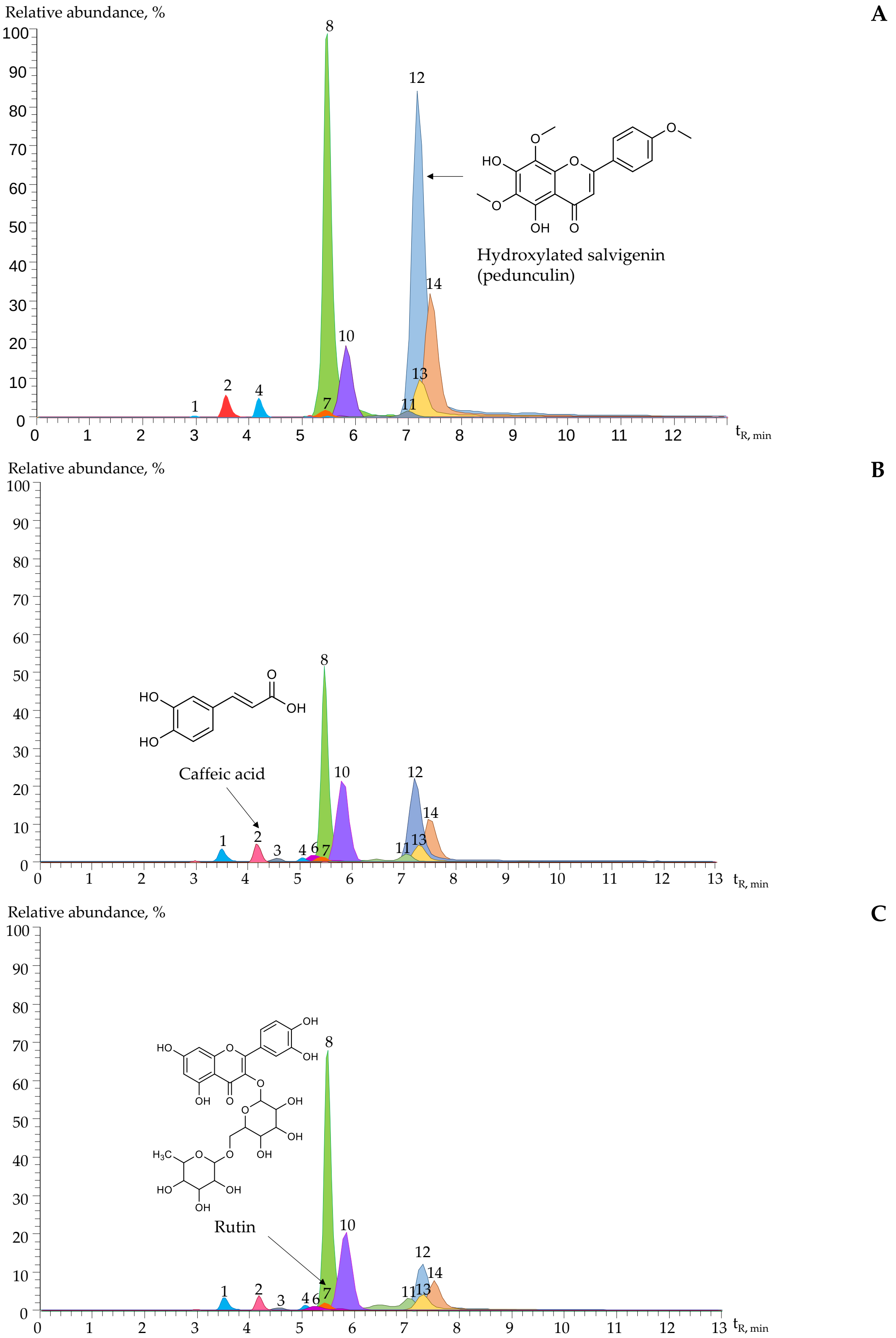

Figure S5. Extracted ion currents from protonated [M+H]$^+$ or deprotonated [M–H]$^-$ molecules, obtained by UHPLC-ESI$^{+/-}$-Orbitrap-MS (scale 6.2 x 10^7), of compounds in the hydroalcoholic extracts of *H. colombiana*, isolated from postdistillation residues, from plants collected at different phenological stages. (**A**) Vegetative stage. (**B**) Flowering stage. (**C**) Post-flowering stage. Compound identification appears in Table S5.

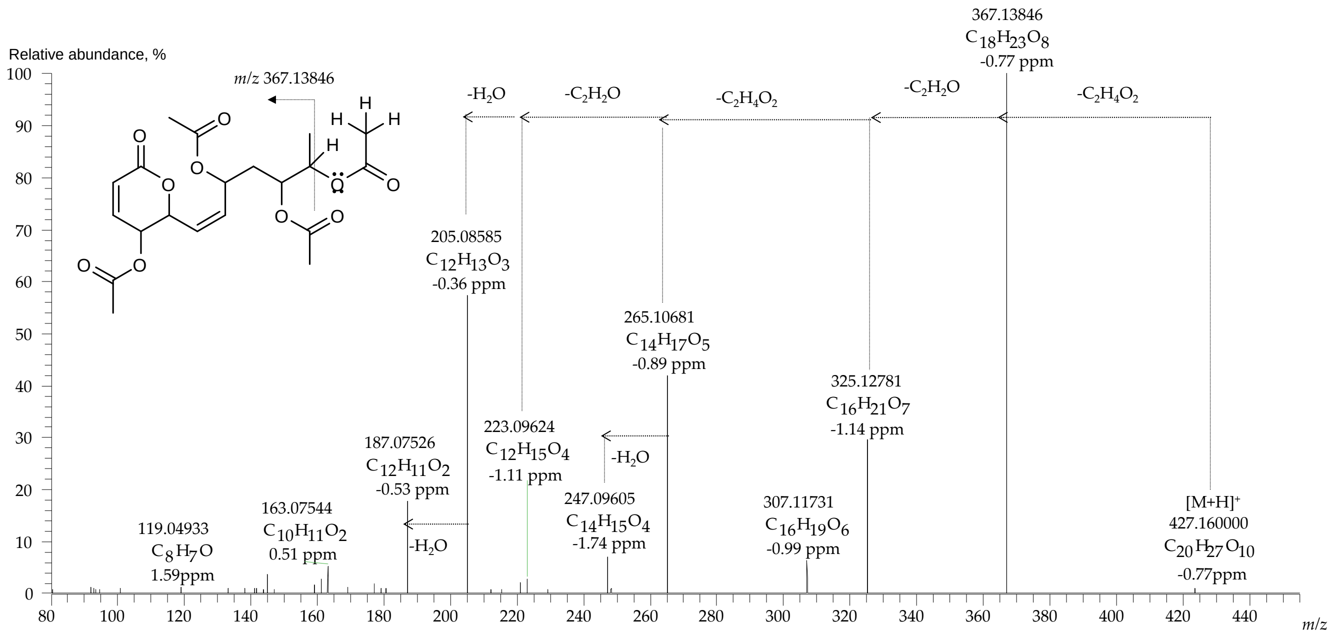

Figure S6. Mass spectrum of the protonated $[M+H]^+$ molecule with *m/z* 427.16000, a pyranone, obtained by the selected ion monitoring mode (SIM), with HCD 10 eV.
